# Selenomethionine against titanium particle-induced osteolysis by regulating the ROS-dependent NLRP3 inflammasome activation via the β-catenin signaling pathway

**DOI:** 10.3389/fimmu.2023.1171150

**Published:** 2023-07-20

**Authors:** Ruixuan Yu, Yongjian Yuan, Zhicheng Liu, Long Liu, Zhaoning Xu, Yunpeng Zhao, Chunwang Jia, Pengfei Zhang, Hang Li, Yuhao Liu, Yi Wang, Weiwei Li, Lin Nie, Xuecheng Sun, Yuhua Li, Ben Liu, Haichun Liu

**Affiliations:** ^1^ Department of Orthopaedics, Qilu Hospital of Shandong University, Jinan, Shandong, China; ^2^ The First Clinical Medical School, Shandong University, Jinan, Shandong, China; ^3^ Department of Pathology, Qilu Hospital, Cheeloo College of Medicine, Shandong University, Jinan, Shandong, China; ^4^ School of Nursing and Rehabilitation, Shandong University, Jinan, Shandong, China; ^5^ Department of Plastic and Burns Surgery, The Second Hospital of Shandong University, Jinan, Shandong, China; ^6^ Emergency Medicine Center, The Second Hospital of Shandong University, Jinan, Shandong, China; ^7^ Department of Orthopedic Trauma, Weifang People’s Hospital, Weifang, Shandong, China

**Keywords:** selenomethionine, titanium particle-induced osteolysis, β-catenin, inflammatory osteolysis, NLRP3

## Abstract

Wear debris-induced osteolysis, especially titanium (Ti) particles-induced osteolysis, is the most common cause of arthroplasty failure with no effective therapy. Previous studies have suggested that inflammation and impaired osteogenesis are associated with Ti particles -induced osteolysis. Selenium (Se) is an essential trace element in the human body, which forms selenomethionine (Se-Met) in nature, and selenoproteins has strong anti-inflammatory and antioxidant stress effects. In this study, the effects of Se-Met on Ti particles-induced osteolysis were observed and the potential mechanism was explored. We found that exogenous Se-Met relieved osteolysis induced by Ti particles in two animal models and MC3T3-E1 cells. We found that the addition of Se-Met effectively inhibited Ti particle-induced inflammation by regulating reactive oxygen species-dependent (ROS-dependent) NOD-like receptor protein 3 (NLRP3) inflammasome activation. These therapeutic effects were abrogated in MC3T3-E1 cells that had received a β-catenin antagonist, suggesting that Se-Met alleviates inflammatory osteolysis via the β-catenin signaling pathway. Collectively, these findings indicated that Se-Met may serve as a potential therapeutic agent for treating Ti particle-induced osteolysis.

## 1 Introduction

Total joint arthroplasty (TJA) is one of the most effective surgical procedures for treating terminal rheumatic arthritis and severe osteoarthritis, providing pain relief and improving the patients’ quality of life. As the aging population is growing worldwide and the number of patients undergoing this surgery is increasing each year, the demand for TJAs is predicted to increase substantially (1). Therefore, the side effects of TJAs during long-term follow-up are a major concern, especially wear debris-induced osteolysis, which is considered to trigger implant loosening and affect the longevity of the implant (2, 3). It is mainly caused by a chronic inflammation reaction triggered by debris, including Ti particles (4), and disrupts bone homeostasis by stimulating various cell types, such as osteoblasts, osteoclasts, macrophages, and fibroblasts, to secrete proinflammatory cytokines, including IL-6, IL-1β, and tumor necrosis factor alpha (TNF-α) (5). Bone homeostasis is based on the balance between osteogenesis and bone resorption, and previous studies have focused on increased osteoclastic bone resorption (6), which has been mainly related to osteoclasts and macrophages (7), but the effect of osteoblasts is of great importance.

Se is an essential trace element in the human body and has been proven to exert biological functions in various systems (8), and Se-Met is one of the main natural forms of Se in living organisms (9). Selenoproteins is considered of great importance in inflammation and immunity, adequate levels of Se are necessary for initiating immunity (10), and previous studies have shown that Se treatment enhances the osteoblastic differentiation of bone marrow stromal cells (BMSCs) and inhibits the differentiation and formation of mature osteoclasts (11), facilitating osteogenic differentiation and bone healing. Selenium nanoparticles were recently found to suppress NLRP3 inflammasome activation in acute kidney injury mouse model (12), while a Se-deficient diet can suppress the expression of selenoprotein, which has an anti-oxidant function, and causes mitochondrial dysfunction and apoptosis of chondrocytes (13). Considering the physiological role of Se and selenoproteins in anti-oxidation and the detrimental effects of oxidative stress and inflammation in wear debris-induced osteolysis, whether Se can play a role in wear debris-induced inflammatory reactions and osteogenic inhibition remains unknown.

The NLRP3 inflammasome is a critical component of the innate immune system (14), and several studies have revealed that the NLRP3 inflammasome contributes to the pathogenesis of wear debris mediated inflammatory osteolysis (15) and is related to the induction of inflammatory mediators such as prostaglandin E2 (PGE2), TNF-α, and IL-1β (16), and Ti particle-induced reactive oxygen species production and structural changes in the mitochondria have been verified (17), thus providing proof of concept that pharmacological inhibition of the NLRP3 inflammasome is a viable therapeutic strategy. It has been reported that inflammation and osteogenesis inhibition are attenuated through activation of the β-catenin signaling pathway in Ti particle-induced osteolysis (18), and Se-Met has been shown to alleviate many inflammatory reactions (19–21).

This study aimed to investigate the role of Se-Met in Ti particle-induced osteolysis. In this study, we plan to determine the function of Se-Met in Ti particle-induced osteolysis in animals models and MC3T3-E1 cells, and we will explore the potential involvement and underlying mechanisms of NLRP3 inflammasome in the Ti particle-induced osteolysis following Se-Met treatment.

## 2 Methods

### 2.1 Preparation of Ti particles

**Figure 1:**
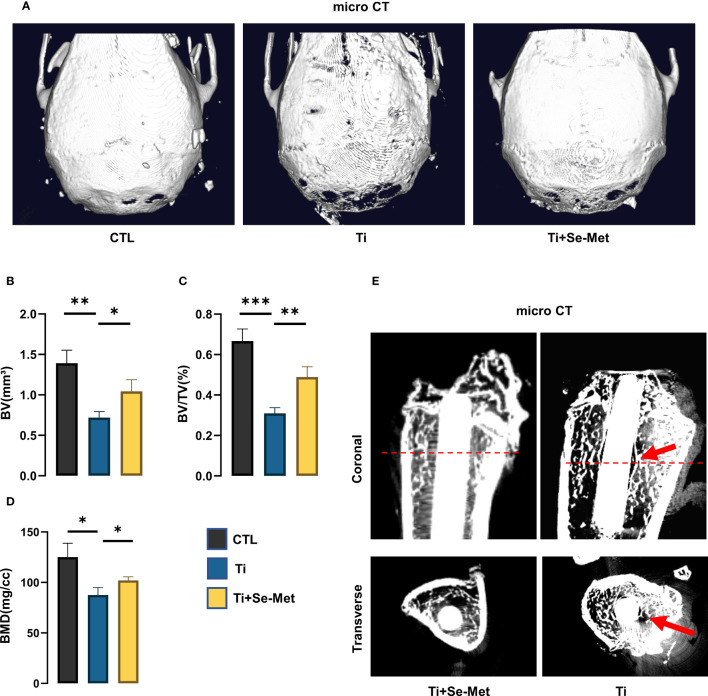
Se-Met attenuated Ti-particle-induced osteolysis. **(A)** Representative images of 3D reconstruction of mouse cranial bones; **(B-D)** quantitative analysis of BV, BV/TV (%), BMD (mg/cc); **(E)** Representative images of rat femurs, identified with red arrows showing osteolysis area. *P < 0.05, **P < 0.01, ***P < 0.001.

Ti particles (catalog #IRMM531A) were purchased from Sigma Corporation (Sigma, St. Louis, MO, USA) and scanned using a scanning electron microscope, as shown in **Figure 1E**. The sizes and distributions of the Ti particles have been previously measured and reported. The average size of the Ti particle was 3.31 ± 2.38 μm. Ti particles were soaked in 75% alcohol for 24 h and then rinsed thrice with sterile water, heated at 180° for 8 h. Then, the Ti particles were immersed in sterile PBS. The imulus assay (LAL, Biowhittaker, USA) was used to detect the liquid in which the particles were soaked to ensure that the endotoxin levels were under 0.3 EU/ml. Titanium rods, 10 mm in length and 1 mm in diameter, were purchased from Sigma (St. Louis, MO, USA). After cleaning the rods thrice with 75% alcohol, the rods were sterilized by autoclaving and subsequently placed under sterile conditions until use.

### 2.2 Animals and Ti particles–induced osteolysis model

The experimental procedures were approved by the institutional Animal Care and Use Committee of Shandong University, and met the guidelines for the Care and Use of Laboratory Animals. Ten-week-old C57BL/6 wild-type (WT) mice were provided by the Experimental Animal Center of Shandong University. Twelve-week-old male Sprague-Dawley (SD) rats were provided by Vital River (Beijing, China). In summary, we constructed two animal models.

#### 2.2.1 Cranial osteolysis models

The method used to establish cranial osteolysis models in mice has been described in our previous study. WT mice were divided into three groups (n = 7 per group): sham control group (CTL group), Ti particle only group (Ti group), and Ti particles + SE-MET treatment group (SE-MET group). All mice received an intraperitoneal (I.P.) injection of 1% pentobarbital sodium anesthesia at a dose of 50 mg/kg before surgery. Subsequently, each mouse underwent a 0.5-cm sagittal incision, and the periosteum remained intact. To simulate Ti particle-induced osteolysis, 100 μl of 0.1 mg/ml titanium particle suspension was injected directly into the skull and periosteum using a sterile technique in the Ti group and Se-Met treatment group. In contrast, in the sham group, no other intervention was performed, and the full-thickness skin was then sutured. Se-Met (Cat: HY-B1000) was purchased from MCE (Monmouth Junction, NJ, USA), and it was dissolved in sterilized treated drinking water to make an aqueous solution of 1mg/L. The SE-MET group (both rats and mice) was treated with 1 mg/L SE-MET solution instead of normal drinking water, and the mice in the CTL and Ti groups were treated with normal drinking water. The duration of Ti particle-induced osteolysis induction was 4 weeks (22).

#### 2.2.2 Intramedullary nail osteolysis models

Fourteen SD rats with an average weight of 300 ± 30 g were randomly divided into two groups (n = 7 per group): the Ti particle only group (Ti group) and the Ti particles + SE-MET treatment group (SE-MET group). Before surgery, all rats were anesthetized by I.P. injection of 1% sodium pentobarbital at a dose of 40 mg/kg. The knee joint was disinfected and the knee capsule was dissected until the articular surface was exposed. A hole was punched in the intercondylar fossa. Before the Ti rod was implanted, 100 μL of 0.1 mg/ml Ti particle suspension was injected into the bone marrow cavity, and then the rod was inserted into the hole. Residual Ti particles in the joint cavity were cleaned with sterile saline and then closed (23).

### 2.3 Micro-CT Assessment

Femurs with Ti rods and calvarial tissues were initially fixed in formalin and subsequently examined by micro-CT with a Sky-scan1176 scanner and associated analysis software (SkyScan, Aartselaar, Belgium). The scanning parameters were set as follows: 18 μm per layer, under a voltage of 50 kV and a current of 500 μA. Cone Beam Reconstruction software (SkyScan) was used for the reconstruction of three-dimensional (3D) images and to evaluate different histomorphometric measurements: BMD (mg/mm2), BV (mm3), and BV/TV (%).

### 2.4 Histological and immunohistochemical analysis

Paraffin-embedded sections were prepared. Morphological characteristics of the rat femurs were observed using hematoxylin and eosin (H&E) staining. Masson staining (Salorbio, China) was used to stain new immature collagen in the blue zone by tissue coloration. Immunohistochemical staining for osteocalcin (OCN) (ab93876, Abcam, USA), COX2 (ab179800, Abcam, USA), and NLRP3 (ab263899, Abcam, USA) was also performed. Images were obtained using a light microscope.

### 2.5 Cell culture

MC3T3-E1 cells were obtained from the Cell Bank of the Chinese Academy of Sciences (Shanghai, China). MC3T3-E1 cells were maintained in α-MEM (Gibco, Brooklyn, NY) containing 10% FBS, 100 μg/ml streptomycin, and 100 U/ml penicillin in a 5% CO2 humidified atmosphere at 37°C, adherent cells were cultured until the cells reached approximately 90% confluence. The osteogenic medium was α-MEM supplemented with 10% FBS, 10 mM β-glycerophosphate, 0.5 mM vitamin C, and 0.1 μM dexamethasone. The induction time of Osteogenesis was induced for 14 d. Osteolysis was induced by adding 10 μg/cm^2^ Ti particles. Cells were cultured in 6-well plates at a density of 1 × 10^5^ cells/well in an incomplete medium and stimulated with 10 μg/cm^2^ Ti particles solution, with or without Se-Met (5 μM) for 24 h. The culture medium and cells were collected for further experiments.

### 2.6 CCK-8 assay

To perform the Cell Counting Kit-8 (CCK-8) assay, MC3T3-E1 cells were seeded in 96-well plates at a density of 5000 cells per well. After allowing the cells to adhere for 12 h, different concentrations of the experimental drug were added to the wells and incubated for 24 h. Following this, 10μL of CCK-8 reagent was added to each well and incubated for another 15 min. The absorbance was measured at 450nm using a microplate reader. The data obtained was then used to calculate cell viability and compare the effects of the experimental drug on cellular proliferation.

### 2.7 Red S staining

After being cultured in osteogenic medium for 2 weeks, MC3T3-E1 cells were washed with PBS, fixed in 4% paraformaldehyde for 20 min, and stained for 30 min with 1% Alizarin Red Staining Solution (Servicebio. Nanjing. China). After the staining, the remaining dye solution was rinsed with distilled water. Stained calcified nodules were observed and photographed under a light microscope.

### 2.8 ALP staining

To assess Alkaline phosphatase (ALP) activity, MC3T3-E1 cells were cultured in osteogenic medium for a week and then fixed in 4% paraformaldehyde for 15 minutes. Following this, the cells were rinsed three times with PBS and stained with BCIP/NBT working solution in the dark for 60 minutes. Microscopic analysis was performed to examine the staining results.

### 2.9 Protein isolation and Western blotting analysis

Total protein was isolated from MC3T3-E1 with RIPA Lysis Buffer (Servicebio Corporation, Nanjing. China). Then the total protein was centrifuged at 12000 RPM for 6 min at 4°, and protein concentrations were measured using the BCA Protein Assay Kit (Beyotime Biotechnology Corporation). Equal amounts of the extracted proteins were electrophoresed on SDS-PAGE gels and transferred onto polyvinylidene difluoride membrane. Subsequently, 5% BSA was used to block the membranes for 1 h at room temperature and membranes were then washed thrice in TBST and incubated with primary antibodies overnight at 4°C, including anti-NLRP3 (1:1000 dilution, SC06-23, ThermoFisher Corporation, USA), anti-iNOS (1:1000 dilution, ab15323, Abcam Corporation, USA), anti-COX2 (1:2000 dilution, 12375-1-AP, Proteintech Corporation, USA), anti-COL1 (1:1000 dilution, 14695-1-AP, Proteintech Corporation, USA), anti-Osteopontin (OPN) (1:1000 dilution, ab214050, Abcam Corporation, USA), anti-RUNX2 (1:1000 dilution, #12556S, Cellsignal Corporation, USA), anti-β-Catenin (1:1000 dilution, 17565-1-AP, Proteintech Corporation, USA), anti-β-tubulin (1:2000 dilution, 10094-1-lg, Proteintech Corporation, USA), and anti-GADPH (1:2500 dilution, ab9485, Abcam Corporation, USA). After washing thrice with TBST buffer, the membranes were incubated with HRP-conjugated anti-goat IgG, anti-mouse IgG, or anti-rabbit IgG as secondary antibodies. The DNR Bio-Imaging System was used to detect protein levels on the membrane.

### 2.10 Real-time quantitative PCR

Total mRNA was isolated from MC3T3-E1 cells using the TRIzol reagent. Complementary DNA was synthesized from total mRNA using the PrimeScript RT Reagent Kit. RNA PCR kit (Servicebio. Nanjing. China) was used for the real-time PCR analysis. All procedures were performed per the manufacturer’s instructions. **Table 1** lists the PCR primer sequences.

**Table 1:** Primers used for quantitative real-time PCR.

Source	Target	Forward primer, 5’-3’	Reverse primer, 3’-5’
Mouse	COX-2 iNOS RUNX-2 Caspase-3 β-Catenin GADPH	GGAACTTTCTGGTCCCTTCAG GCCAAGCTGAAATTGAATGAGGA TTGACCTTTGTCCCAATGC CTCGCTCTGGTACGGATGTG ACGGTGCCGCGCCGCTTATA AGCAGTCCCGTACACTGGCAAAC	TGTGTTTGGAGTGGGTTTCA TTCTGTGCCGGCAGCTTTAAC AGGTTGGAGGCACACATAGG TCCCATAAATGACCCCTTCATCA TAGCCATTGTCCACGCAGCGG TCTGTGGTGATGTAAATGTCCTCT

### 2.11 Immunofluorescence

MC3T3-E1 cells were seeded on coverslips containing osteogenic medium in 24-well plates. The cells were then fixed with 4% paraformaldehyde and permeabilized with 0.2% Triton X-100 (Servicebio. Nanjing. China) for 15 min. They were then blocked with a blocking buffer for 60 min. Next, primary antibodies, including anti-NLRP3 (1:50), anti-COX2 (1:150), anti-OCN (1:200), and anti-β-catenin (1:400), were added to each well and incubated at 4°C for 12 h. Subsequently, the cells were rinsed and incubated for 60 min in the dark with the corresponding secondary fluorescent antibodies (Alexa Fluor^®^488 or 647 (Abcam). Fluorescently stained cells were counterstained with DAPI for 15 min. Cover slides were placed on microscope slides with a fluorescent anti-fade solution (Beyotime) and observed under a fluorescence microscope (Zeiss). Fluorescence intensity was measured using ImageJ software.

### 2.12 Flow cytometry

Briefly, Mc3t3-e1 cells from each group were analyzed using flow cytometry. Cells were stained with annexin V-FITC and propidium iodide for 20 min at room temperature in the dark, according to the manufacturer’s protocol. Apoptosis was detected by CytoFLEX S flow cytometry (Beckman Coulter, Indianapolis, IN, USA). The measured data were analyzed using FlowJo software. The measured data were analyzed using FlowJo software.

### 2.13 ROS assay

To detect intracellular ROS, we used a ROS assay kit. All procedures were performed per the manufacturer’s instructions. Briefly, after washing twice with sterile PBS, MC3T3-E1 cells were stained with 10 μM DCFDA at 37°C for 20 min in the dark and mixed every 4 min. The MC3T3-E1 cells were then washed with serum-free culture medium thrice to reduce interference from excess DCFDA. DCFDA fluorescence intensity in each group was measured using an LSM780 laser scanning confocal microscope (ZEISS, Germany).

### 2.14 Transmission electron microscopy

MC3T3-E1 cells were collected by trypsinization, transferred into 2 mL centrifuge tubes, and fixed with fixative solution (Servicebio, G1102) for 2 h at 4°C. Cells were post-fixed in 1% osmium tetroxide in 0.1 M phosphate buffer (pH 7.4) for 2 h at room temperature (20°C). MC3T3-E1 cells were dehydrated in a graded ethanol series (50%, 70%, 80%, 90%, 95%, 100%, and 100%) for 15 min, and then infiltrated into the embedding solution with propylene oxide overnight. Ultrathin sections (50 nm) were obtained using an EM UC7 ultramicrotome, post-stained with uranyl acetate and lead citrate, and visualized using transmission electron microscopy (24).

### 2.15 Enzyme-linked immunosorbent assay

After culturing MC3T3-E1 cells with different interventions, the medium was collected for enzyme-linked immunosorbent assay (ELISA). IL-1β levels were assayed by ELISA using a commercial kit (Elabscience, Wuhan, China) according to the manufacturer’s instructions.

### 2.16 RNA-seq

The total RNA of MC3T3-E1 cells was obtained using TRIzol Reagent. The extracts were screened, amplified by PCR, and sequenced using an MGI T7 instrument. For bioinformatics analysis, raw reads that contained the adapter or had low quality (Q-value ≤20) were deleted and then located in the mouse-related genome using HISAT2 (version 2.1.0). Samtools was used to sequence the resulting files, and HTSeq (version 0.9.0) analysis was performed to determine the count of each gene. Gene Ontology (GO) and Kyoto Encyclopedia of Genes and Genomes (KEGG) pathway analyses were performed.

### 2.17 Statistical analysis

The data are presented as the mean ± standard deviation and were analyzed using GraphPad Prism (GraphPad Software Inc., USA). The software was used for the statistical analyses. Student’s t-test or one-way analysis of variance (ANOVA) was used to determine the statistical significance of differences. Statistical significance was set at P < 0.05

## 3 Results

### 3.1 Se-Met suppresses the severity of Ti particle-induced osteolysis *in vivo*

Ti particle-induced osteolysis has been widely reported in the literature. To investigate the role of Se-Met in Ti particle-induced osteolysis, we established a cranial osteolysis model in mice and an intramedullary nail osteolysis model in rats. We then performed CT scans on both models and analyzed the 3D reconstruction images of the mouse cranial bone lysis model, which showed that the periprosthetic bone mass was significantly damaged in the titanium group, the cranial thickness was thinner than that in the sham control group, and the bone mass was significantly destroyed (**Figure 1A**). However, after the intervention with Se-Met, the bone loss was alleviated significantly, and the quantitative analysis of bone parameters (**Figures 1B–D**) showed that BV/TV, BMD, and BV were significantly higher than those in the Ti group. In the 2D reconstructed images of the rat intramedullary nail osteolysis model, osteolysis around the Ti rod was evident. Compared to the Ti group, osteolysis was significantly reduced in the Se-Met treatment group (**Figure 1E**).

**Figure 2:**
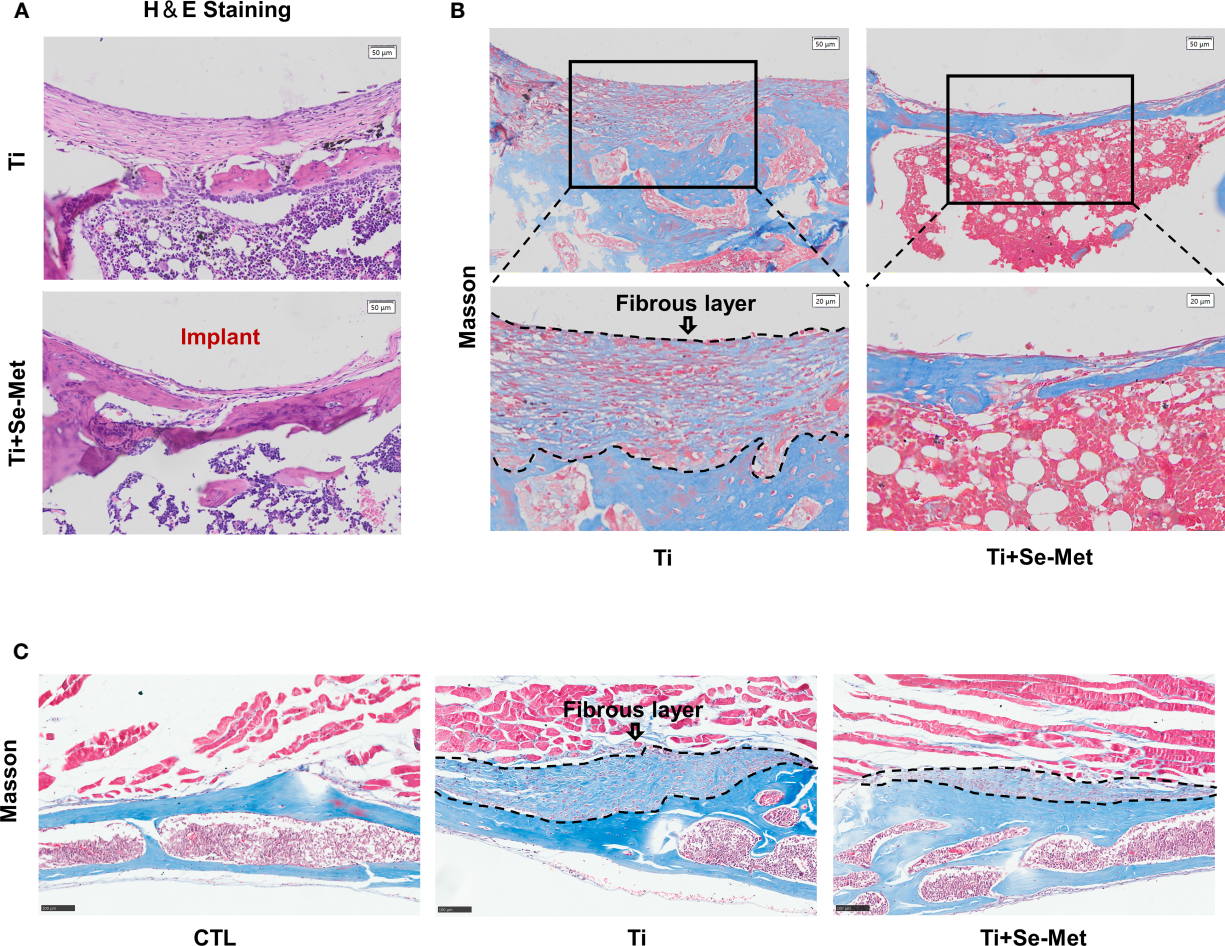
Se-Met rescued Ti-particle-induced osteolysis. **(A)** H&E staining of rat femurs; **(B, C)** Masson staining of rat femurs and mouse cranial bones, the areas marked by black dashed lines are inflammatory fibrous layers.

Consistently, H&E and Masson staining and immunohistochemistry confirmed the suppression of periprosthetic osteolysis by treatment with Se-Met in Ti particle-induced osteolysis in mouse and rat models. H&E staining of rat femurs showed absorption of bone structure in the Ti group, and inflammatory infiltration was found in the bone absorption area, while the bone destruction areain the Se-Met group was significantly decreased, and the number of inflammatory cells was also reduced (**Figure 2A**). To further explore the components of inflammatory infiltration, Masson staining of rat femurs showed a large amount of disordered fibrous tissue in the inflammatory infiltration area of osteolysis (**Figure 2B**), which was restored by adding Se-Met. Masson’s trichrome staining was performed using the mouse osteolysis model. It was confirmed that Se-Met alleviated inflammatory fibrous tissue infiltration induced by Ti particles (**Figure 2C**).

### 3.2 Effects of Se-Met on inhibition of inflammatory osteolysis *in vivo*

**Figure 3:**
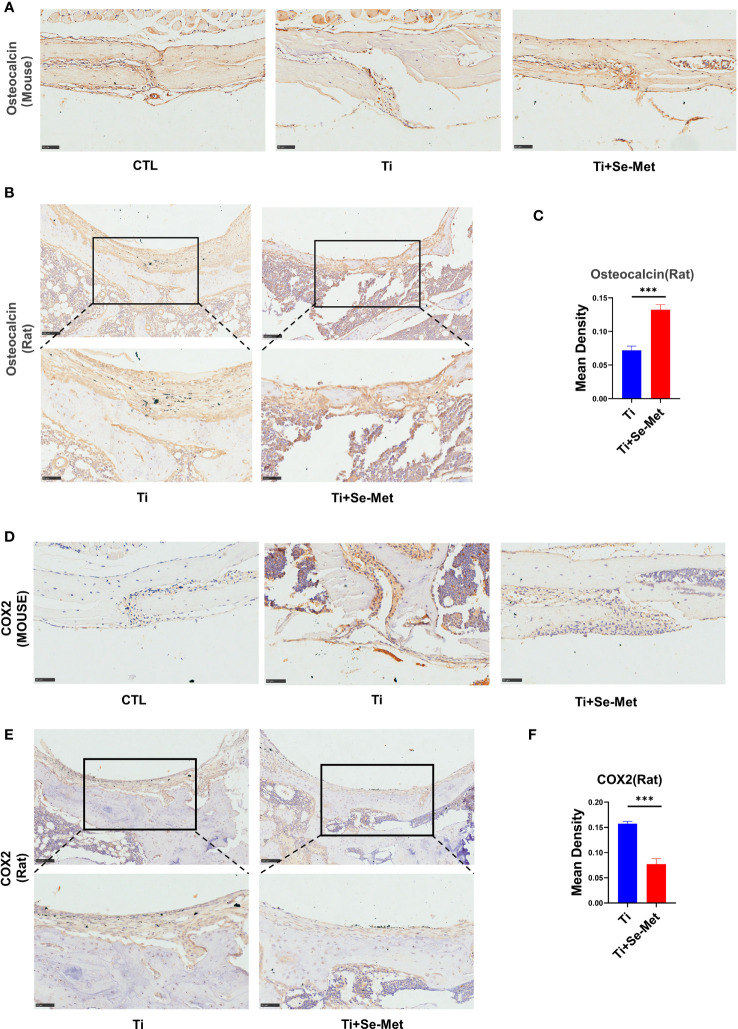
Se-Met rescued Ti-particle-induced osteolysis in Immunohistochemistry. **(A-C)** Immunohistochemical results showed that Se-Met rescued the decreased secretion of OCN induced by Ti particle stimulation; **(D-F)** The results of immunohistochemistry showed that Se-Met inhibited the increase of COX2 expression induced by Ti particles ***P < 0.001.

To test the hypothesis that Se-Met may have an inhibitory role in periprosthetic osteolysis, immunohistochemical staining was performed for each group in the mouse skulls. The results showed that the expression of OCN in the Ti particle group was reduced, which could be completely reversed after treatment with Se-Met (**Figure 3A**). The same result was observed in the OCN staining of rat femurs (**Figures 3B, C**). According to previous studies, inflammatory responses play a critical role in Ti particle-induced osteolysis, and inhibition of inflammation in osteoblasts can exert a positive effect on osteolysis. We performed immunohistochemical staining of COX2 and found that the expression of COX2 in the Ti group of mouse skulls was significantly increased compared with the CTL group, while it was reduced in the Se-Met treatment group (**Figure 3D**), and similarly in the COX2 staining of rat femurs (**Figures 3E, F**).

### 3.3 Se-Met attenuates inflammatory osteolysis induced by Ti particles *in vitro*

**Figure 4:**
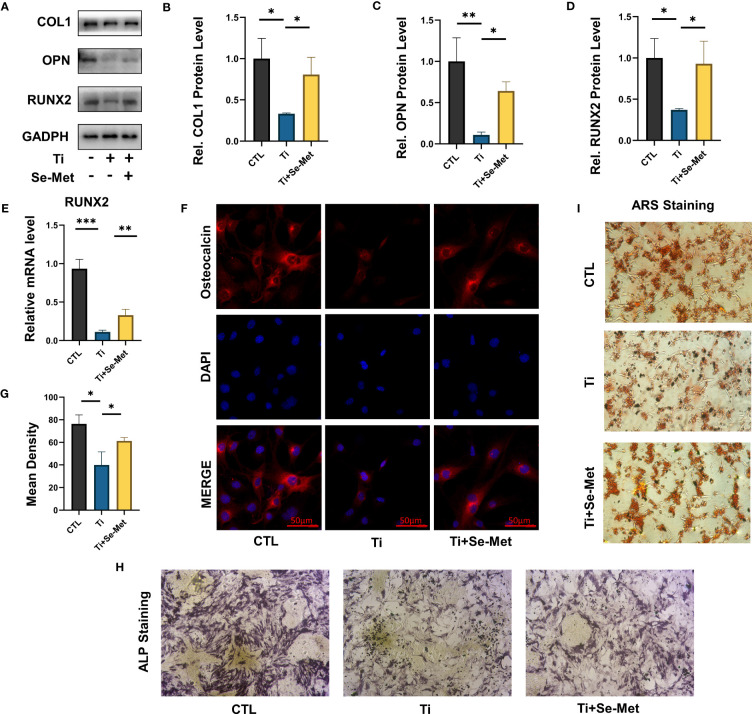
Se-Met has a protective effect on osteoblast dysfunction induced by Ti particles. **(A-D)** Western blot results showed that Se-Met significantly rescued the decreased expression of osteoblast-related factors Col 1, RUNX-2, and OPN caused by Ti particles stimulation; **(E)** Real-time PCR results showed that Se-Met effectively relieves the RUNX2 expression decreased caused Ti particles; **(F, G)** Se-Met treatment rescued Ti particle-induced OCN expression in MC3T3-E1 cells, as assayed by cell immunostaining; **(H)** Representative images of ALP staining at 7 d; **(I)** Representative images of ARS staining at 21 d. * P < 0.05, **P < 0.01, ***P < 0.001.

As mentioned above, we found that Se-Met rescued osteogenesis inhibition and reduced local inflammation caused by Ti particles *in vivo*, to further verify the conclusion we conducted *in vitro* experiments, osteoblast-related factors, including RUNX-2, OPN, and COL 1, were examined through western blotting, the results showed that osteogenic markers were significantly upregulated in the Se-Met treated group compared with the Ti group, indicating that Se-Met treatment alleviated the Ti particle-induced osteogenesis reduction (**Figures 4A–D**). In addition, the real-time PCR results were consistent with those of western blotting (**Figure 4E**). Immunofluorescence detection showed that OCN expression in the Ti group was significantly lower than that in the CTL group, whereas OCN expression in the Se-Met group was higher than that in the Ti group, which was consistent with the above results (**Figures 4F, G**). We induced osteogenic differentiation of MC3T3-E1 cells stimulated with Ti particles. ALP staining was performed after 7 days, and the results showed that Ti particles could reduce the ALP activity *in vitro*, and Se-Met could restore it (**Figure 4H**). Alizarin red staining was performed after 14 d, and the results showed that Se-Met alleviated the reduction in the mineralization rate caused by Ti stimulation (**Figure 4I**).

**Figure 5:**
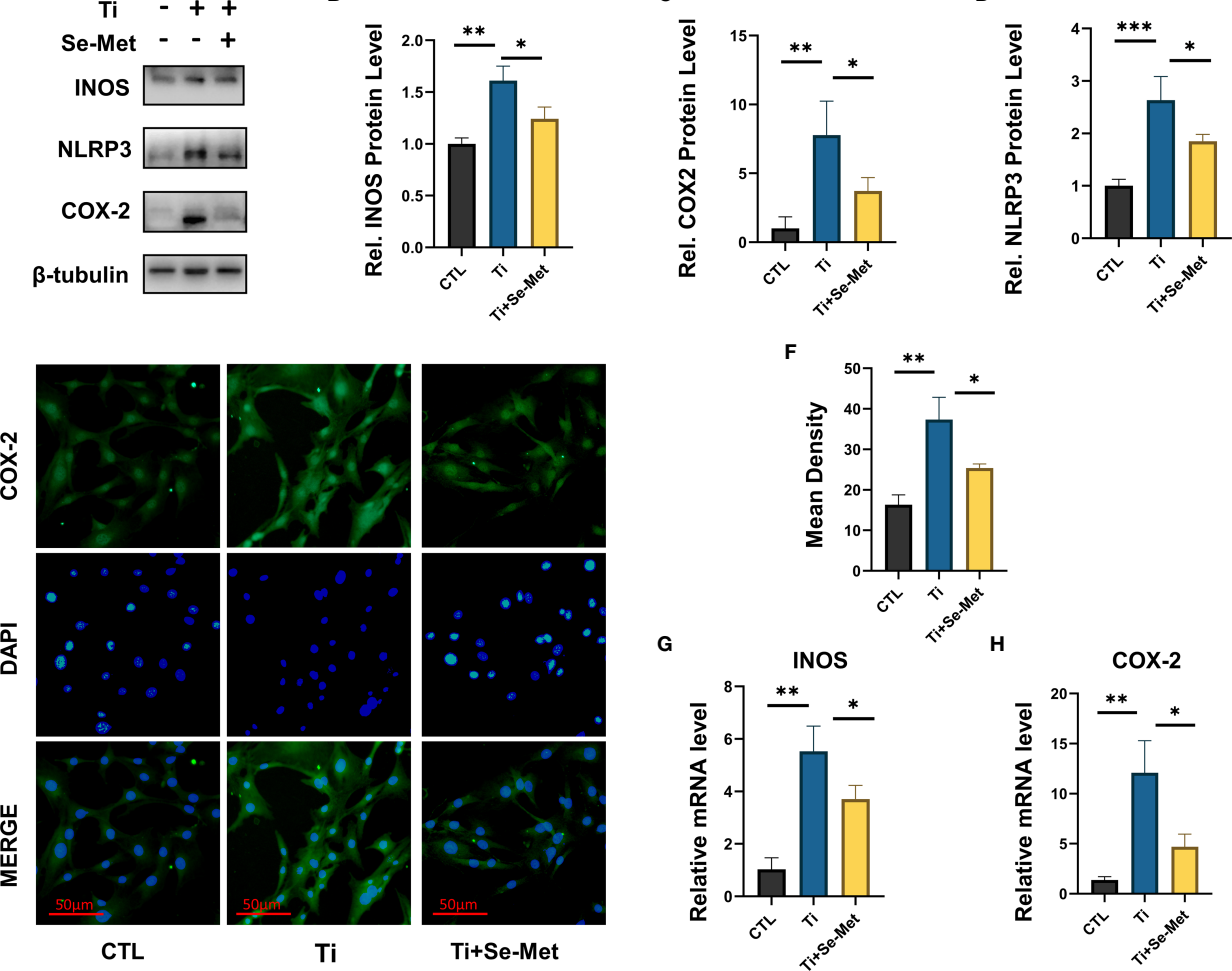
Se-Met has an inhibitory effect on the inflammatory response induced by Ti particles. **(A-D)** Se-Met inhibited the expression of inflammatory cytokines, such as iNOS, COX-2, and NLRP3, which were enhanced with Ti particle stimulation, as indicated by western blot. **(E, F)** Se-Met treatment inhibited Ti particle-induced COX2 expression in MC3T3-E1 cells, as assayed by cell immunostaining. **(G, H)** Se-Met significantly inhibits the upregulation of COX2 and INOS induced by Ti particle stimulation, as shown by real-time fluorescent PCR results. *P < 0.05, **P < 0.01, ***P < 0.001.

It is well known that inhibiting the inflammation extent in osteoblasts can exert a positive effect on osteolysis treatment. To further determine whether Se-Met exerts an effect on osteoblasts in inflammatory osteolysis, MC3T3E1 cells were stimulated with Ti particles and cultured in the presence or absence of Se-Met, proteins were collected for western Blotting assay and the results showed that Ti particles stimulation significantly elevated INOS COX2 expression, especially NLRP3, while the additional application of Se-Met substantially recovered their secretion, indicating a lower severity of inflammatory osteolysis (**Figures 5A–D**), COX2 expression was also detected by Immunofluorescence, and the results showed that the increased COX2 expression caused by Ti particles stimulation could be suppressed by Se-Met treatment (**Figures 5E, F**). mRNA was extracted for qPCR to validate the above results (**Figures 5G, H**).

### 3.4 Se-Met antagonizes mitochondrial ROS-dependent NLRP3 inflammasome activation *in vitro*

**Figure 6:**
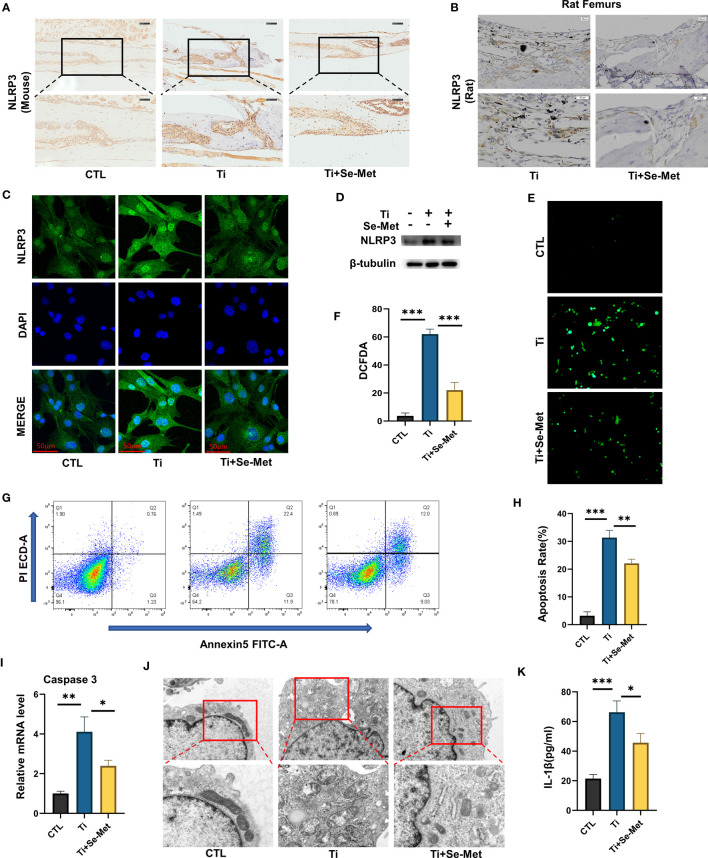
Se-Met antagonizes mitochondrial ROS-dependent NLRP3 inflammasome activation *in vitro*. **(A, B)** Representative images of IHC staining, which showed Se-Met inhibited the up-regulation of NLRP3 expression stimulated by Ti particles *in vivo*; **(C)** Representative images of immunofluorescence staining, which showed treatment of Se-Met inhibited the up-regulation of NLRP3 expression in MC3T3-E1 cells; **(D)** Western blot showed that Se-Met inhibited the rise of NLRP3; **(E, F)** Se-Met antagonized the Ti particle–mediated production of ROS, as detected by the DCFDA assay; **(G, H)** The effect of Ti particles on apoptosis rate of MC3T3-E1 cells was tested by flow cytometry; **(I)** The mRNA were collected from each group, followed by real-time PCR to measure caspase-3 levels; **(J)** Representative TEM images of mitochondria in MC3T3-E1 cells of each group; **(K)** The expression of IL-1β in the culture media of groups of MC3T3-E1, as detected by ELISA. *P < 0.05, **P < 0.01, ***P < 0.001.

Given the fact that inflammatory reactions have detrimental effects on wear debris-induced osteolysis and that the NLRP3 inflammasome plays a critical role in inflammatory responses, we sought to determine whether Se-Met exerts an inhibitory effect on NLRP3 inflammasome activation in inflammatory osteolysis. We evaluated the NLRP3 inflammasome expression in mouse cranial bone through immunohistochemical staining, which showed a significant increase in NLRP3 inflammasome expression in the Ti-stimulated group and a decrease with Se-Met treatment (**Figure 6A**). The rat femur samples also demonstrated that Se-Met reduced the NLRP3 inflammasome expression *in vivo* (**Figure 6B**). The NLRP3 inflammasome expression level *in vitro* was also examined by western blotting, which indicated that NLRP3 inflammasome expression enhancement by stimulation of Ti particles was suppressed by adding Se-Met (**Figure 6C**). We also extracted protein from cells and assayed it by western blotting, which showed that Se-Met treatment reduced the Ti particle-induced elevation in NLRP3 expression (**Figure 6D**). ROS levels were detected using the DCFDA assay (**Figures 6E, F**), which showed that Ti particles enhanced ROS levels, whereas adding Se-Met largely attenuated this effect. Flow cytometry was performed to determine the proportion of apoptotic cells. As shown in (**Figures 6G, H**), the ratio of apoptotic MC3T3-E1 cells increased after stimulation with Ti particles, whereas the addition of Se-Met prevented this effect. Real-time PCR also demonstrated that caspase-3, an apoptosis indicator, was significantly increased by Ti particle stimulation, and se-met treatment alleviated this effect (**Figure 6I**). It is well known that increased ROS generation is closely associated with impaired mitochondrial function. To verify the potential interaction between Se-Met and mitochondrial function, we performed transmission electron microscopy (TEM), high-field images of swollen mitochondria in the MC3T3E1 cells with Ti stimulated showed that the mitochondria surrounded the nucleus, indicating accelerated mitochondrial damage, and these changes were reduced in MC3T3E1 cells with Se-Met treatment (**Figure 6J**). Furthermore, ELISA to detect IL-1β indicated that the enhanced NLRP3 inflammasome production and function induced by Ti particles was alleviated by Se-Met treatment (**Figure 6K**).

### 3.5 Se-Met exerts an effect in inflammatory osteolysis induced by Ti particles through the β-catenin signaling pathway

**Figure 7:**
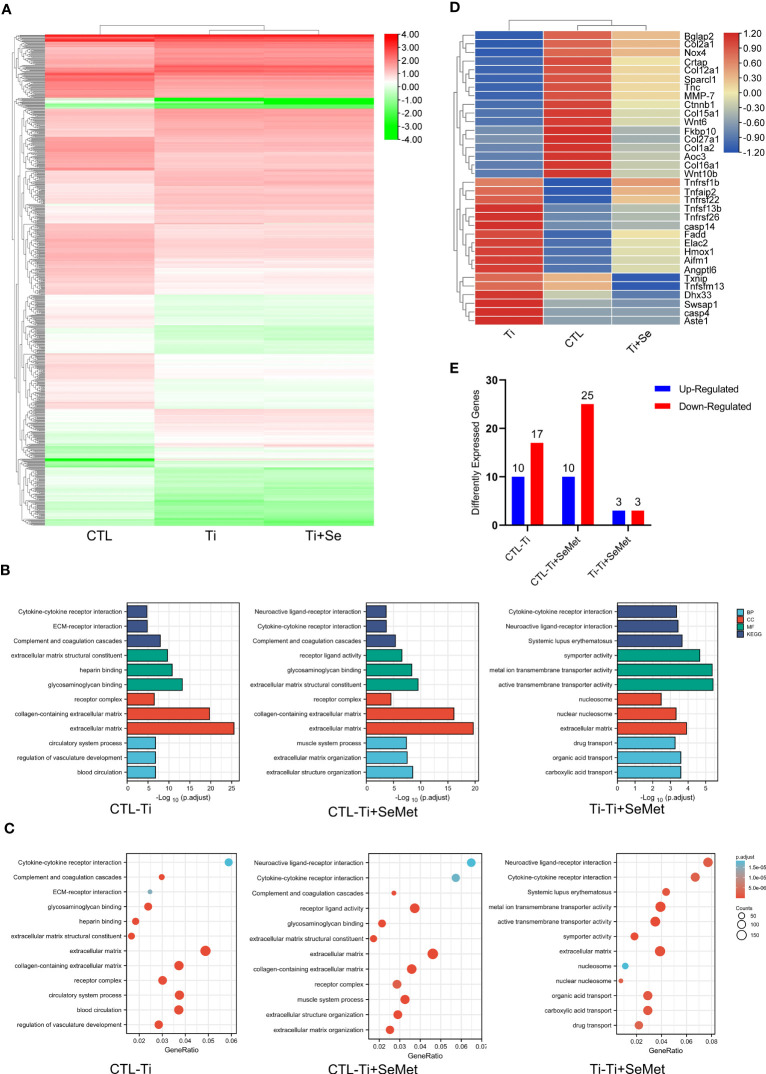
RNA-seq analysis showed that Se-Met rescued osteolysis induced by Ti particles, which was related to the β-Catenin pathway. **(A)** Heat map and cluster analysis; **(B, C)** Significant pathway of GO Enrichment analysis; **(D)** Cluster analysis of osteolysis-related genes; **(E)** Number of differentially expressed novel genes.

**Figure 8:**
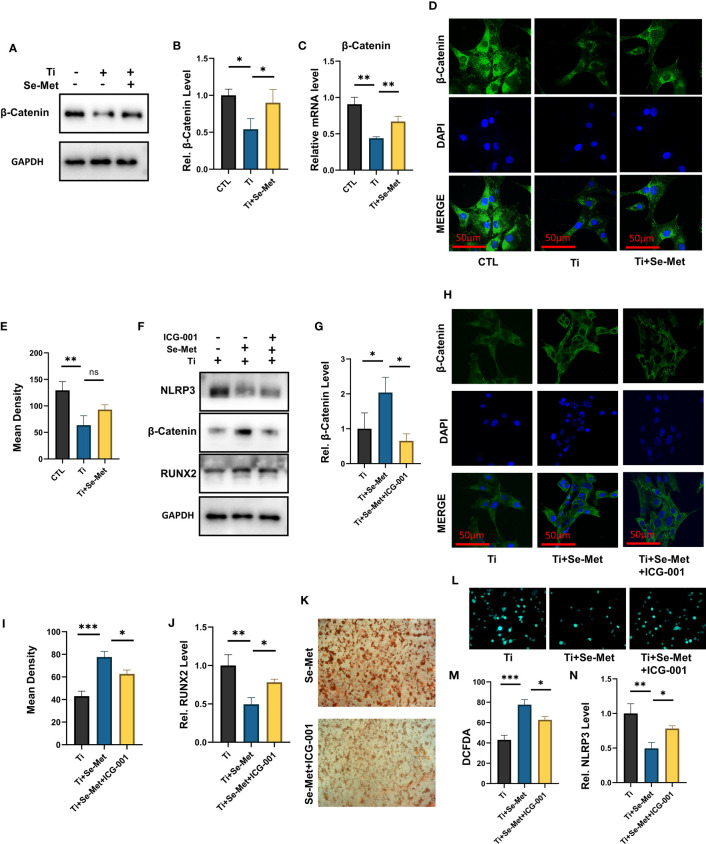
Se-Met antagonizes Ti-particle-induced osteolysis by activating the β-catenin signaling pathway. **(A-C)** The mRNA and total protein were collected from each group, followed by real-time PCR and immunoblotting to measure β-Catenin levels; **(D, E)** Immunofluorescence results showed that Ti decreased the expression of β-Catenin, which was alleviated by Se-Met; **(F)** The total proteins of each group were extracted and detected by Western blot; **(G)** The protective effect of Se-Met against the decrease in β-Catenin induced by Ti stimulation was inhibited by ICG-001; **(H, I)** Immunofluorescence results showed that the addition of SE-met alleviated the decrease of β-Catenin induced by Ti particles, while ICG-001 inhibited this effect; **(J)** WB results showed that the expression of RUNX2 decreased by ICG-001 stimulation; **(K)** Representative images of ARS staining at 21 d, show that ICG-001 inhibited the mineralization rate of MC3T3-E1 cells; **(L, M)** ICG-001 attenuated the ROS inhibitory effect of Se-Met, as detected by the DCFDA assay; **(N)** Western blot results showed that ICG-001 attenuated the down-regulation effect of Se-Met on NLRP3 inflammasome. *P < 0.05, **P < 0.01, ***P < 0.001.

To gain additional insights into the mechanism responsible for the regulation of inflammation and bone formation by Se-Met, we isolated the total mRNA of MC3T3-E1 osteoblasts after stimulation with Ti particles for 24 h, and RNA-seq was analyzed using the MGI T7 platform to generate mRNA profiles. A heat map was plotted, and cluster analyses were performed (**Figure 7A**). The differentially expressed genes (DEGs) were annotated using GO categories (**Figure 7B**) and KEGG pathway analysis (**Figure 7C**). Heat map and cluster analysis of inflammatory osteolysis-related genes showed significant differences between the control group and the Ti particle-stimulated group. The results indicated a decrease in osteogenesis biomarkers in the Ti particle-stimulated group, while the addition of Se-Met prevented this effect, and we noticed increased β-catenin levels in the Se-Met treatment group, which is consistent with the results of real-time PCR and western blotting (**Figures 7D**, **8A-C**). Moreover, the statistics of the number of differential new genes detected are shown in **Figure 7E**. We further performed Immunofluorescence in MC3T3E1 cells, which showed that the inhibition of β-catenin expression by stimulation with Ti particles was relieved by adding Se-Met (**Figures 8D, E**). These data illustrate that β-catenin signaling may be involved in the protective effects of Se-Met on Ti particle-induced osteogenic inhibition.

Bearing in mind that indocyanine green-001 (ICG-001) is a targeted β-catenin signaling inhibitor, we used it to pretreat MC3T3E1 cells. Western blotting showed that ICG-001 eliminated the effect of Se-Met in upregulating β-catenin (**Figures 8F, G**). Immunofluorescence results showed that the addition of SE-met alleviated the decrease in β-catenin induced by Ti particles, while ICG-001 inhibited this effect (**Figures 8H, I**). The results showed that the application of Se-Met substantially enhanced RUNX2 secretion, an osteoblast-related factor, whereas adding ICG001 substantially inhibited this effect (**Figures 8F, J**). Alizarin red staining showed that ICG-001 significantly reduced the mineralization rate of MC3T3E1 cells after 21 d of osteogenic induction (**Figure 8K**). Analysis of ROS levels in each group by the DCFDA assay also showed that ICG-001 eliminated the therapeutic effect of Se-Met (**Figures 8L, M**). Western blotting showed that the inhibitory effect of Se-Met on NLRP3 expression was attenuated by ICG-001 (**Figures 8F, N**). These collective results suggest that ICG-001 blocks the beneficial effects of Se-Met in Ti particle-induced osteogenic reduction through the NLRP3 inflammasome-regulated β-catenin signaling pathway.

## 4 Discussion

Wear debris-induced osteolysis is due to an imbalance in bone homeostasis between bone resorption and formation (25), which involves many cells including macrophages, lymphocytes, fibroblasts, osteoclasts, and osteoblasts (26), Previous research has claimed that bone regeneration inhibition is fundamental in osteolysis initiation (27), and the inflammation response in osteoblasts is demonstrated to be related to attenuated osteogenesis (28), Therefore, treatment of inflammation and promotion of osteogenic differentiation can be an effective method for the prevention and curation of wear debris-induced bone loss after TJA.

Se is an essential trace element in animals and humans and is generally taken up from the diet through food or other forms of external supplementation (29). Plants mainly convert Se into Se-Met and incorporate it into proteins instead of methionine (Met) (9). Selenoproteins are anti-oxidants and can regulat redox balance (10, 30), suggesting that Se-Met might be a candidate for treating wear debris-induced osteolysis. However, whether Se-Met is involved in wear debris-induced osteolysis is poorly understood. This study aimed to demonstrate whether Se-Met represents a promising treatment for alleviating wear debris-induced osteolysis *in vivo*. We found that Ti particle-induced osteolysis was significantly reduced by treatment with Se-Met, which also increased the expression of OCN in femoral slices. We aimed to further explore whether Se-Met mediates its pharmacological effect via suppressing inflammation and osteogenic inhibition in MC3T3E1 cells cocultured with Ti particles. We discovered an enhancement of inflammatory cytokines including INOS COX-2 and NLRP3 inflammasome and downregulation of osteogenic biomarkers including RUNX2 COL-1 and OPN with the addition of Ti particles, the application of Se-Met has been found to exert positive effects in both inhibiting inflammatory cytokines and promoting osteogenesis in MC3T3E1 cells.

The NLRP3 inflammasome plays a detrimental role in inflammation and apoptosis in osteoblasts and contributes to wear debris-induced osteolysis (23), and promoting NLRP3 inflammasome in BMSCs can suppress osteogenic differentiation (31). McCall et al. reported that the functional expression of NLRP3 in osteoblasts is possibly related to apoptotic cell death (32), and previous research has verified the ability of Se-Met to decrease the ROS levels and apoptosis rate in the N2A-SW cell model (33), and mitochondria of cells contribute to the NLRP3 inflammasome activation through several mechanisms. In this study, Se-Met treatment suppressed Ti particle-induced ROS and elevated NLRP3 inflammasome expression in osteoblasts, while inflammatory cytokines and apoptosis were downregulated upon Se-Met treatment. Collectively, these findings strongly suggest that Se-Met inhibits Ti particle-induced apoptosis and inflammation by suppressing the NLRP3 inflammasome.

The β-catenin signaling pathway is closely implicated in osteoblastic differentiation and mineralization (34, 35). Previous research has shown that the β-catenin signaling pathway has a crucial effect on inflammatory osteolysis pathogenesis (36), while Se has been reported to promote the migration and osteogenic differentiation of BMSCs (11), and the ability of Se-Met to reduce oxidative stress has been demonstrated (13). In our study, β-catenin levels decreased after stimulation with Ti particles, which could be upregulated by Se-Met treatment. Intriguingly, Se-Met was shown to elevate the expression of osteogenic biomarkers, including OPN, RUNX2, and COL1, and downregulate the NLRP3 inflammasome and ROS expression. To further explore whether Se-Met exerts the function of promoting osteogenesis by regulating the ROS-dependent NLRP3 inflammasome activation via the β-catenin signaling pathway, ICG-001, a targeted β-catenin signaling pathway inhibitor, was applied to coculture with MC3T3E1 cells. Our study showed that osteoblastic differentiation and bone formation were reduced, and the expression of ROS and NLRP3 inflammasome was elevated, indicating that the rescue effect of Se-Met was alleviated by ICG-001 administration. These results suggest that Se-Met antagonizes the inhibitory effect of Ti particles on bone regeneration with the help of β-catenin signaling pathway activation. The NLRP3 inflammasome is a critical component of the innate immune system that mediates caspase-1 activation and the secretion of proinflammatory cytokines IL-1β/IL-18 in response to cellular damage (14), and Se-Met could regulate the activation of the NLRP3 inflammasome, which suggests the great potential of Se-Met in anti-inflammation treatment. Study has demonstrated that biological macromolecules displayed protective effects against intestinal barrier dysfunction (37), which offer important insights on Se-Met in nonimmunogenicity and safety compared with bioactive molecular mimics.

However, there are some limitations exist in our current study. First of all, metal implants may release metal ions into the surroundings and blood *in vivo*, and their side effects need to be considered (38). However, ultra-high molecular weight polyethylene (UHMWPE) wear particles do not have this effect, and UHMWPE wear particles can be used to establish osteolysis models in subsequent studies (39). Second, we constructed two small animal models and lack of biomechanical analysis. Subsequent studies could be performed in a large animal model to verify the protective effect of Se-Met on osteolysis. Finally, the safety assessment of Se-Met supplementation is lacking, and the safety of ingestion of large amounts of Se-Met for the treatment of osteolysis needs to be further investigated (40).

## 5 Conclusion

**Figure 9:**
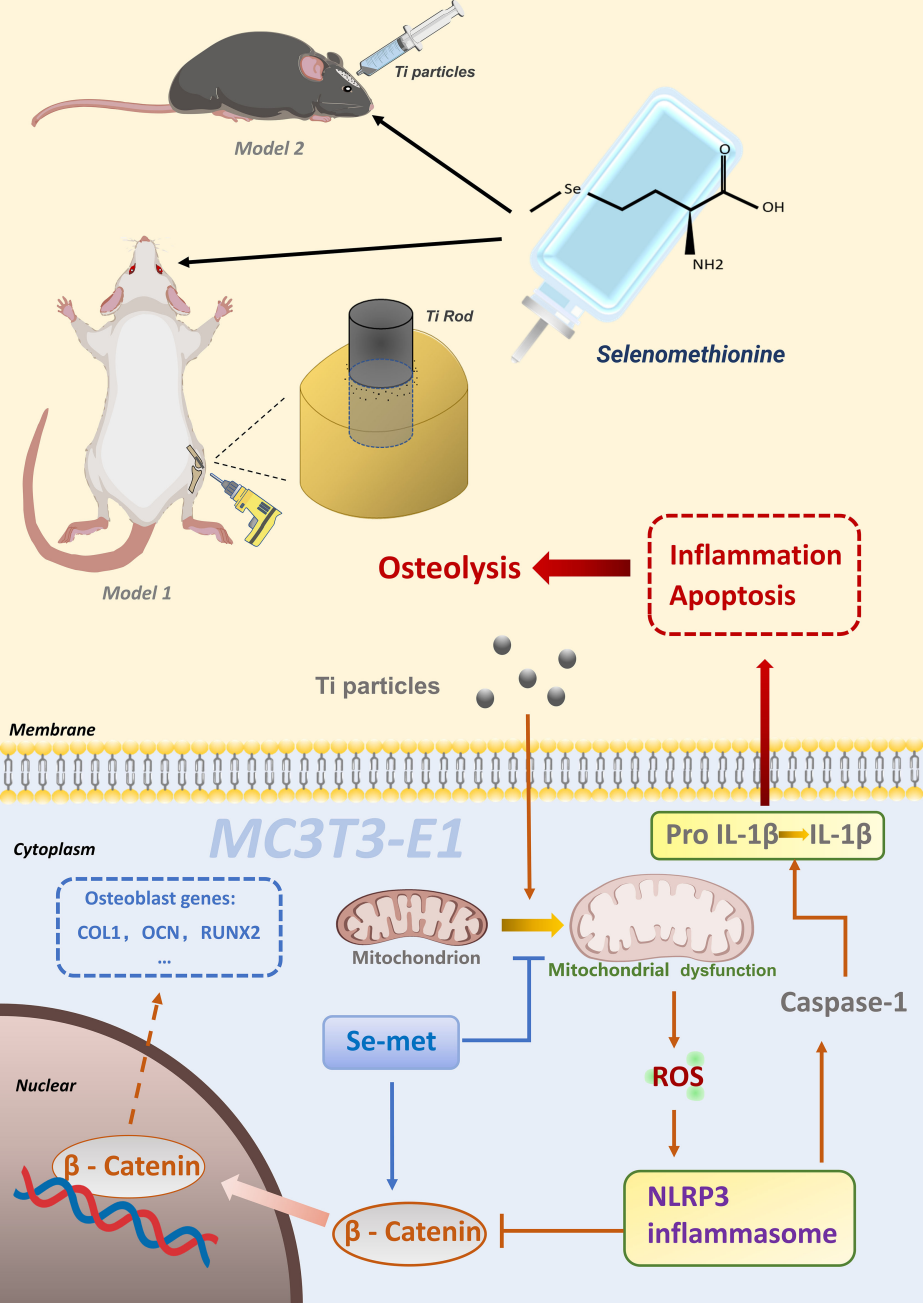
Schematic model of Se-Met in Ti particleinduced osteolysis.

Collectively, it appears that Se-Met plays a protective role in Ti particle-induced disorganization of osteoblasts and impairment of bone formation during osteolysis by suppressing inflammatory cytokine secretion, reducing apoptosis, and promoting bone formation, which might be associated with the activation of the ROS-dependent NLRP3 inflammasome via the β-catenin signaling pathway in osteoblasts (**Figure 9**). In conclusion, the present study sheds light on the prevention and treatment of wear debris–induced prosthetic loosening in the clinic.

## Data availability statement

The data presented in the study are deposited in the GEO repository, accession number GSE236911.

## Ethics statement

The animal study was reviewed and approved by Institutional Animal Care and Use Committee of Shandong University.

## Author contributions

RY, YY, ZL, and LL contributed equally to this work as first authors. They designed and performed experiments, analyzed data, and wrote the manuscript. ZX, YZ, CJ, PZ, HLi, YuhL, YW, WL and LN provided technical assistance in experiments and data analysis. YuhL and BL supervised the project and provided guidance throughout the study. HLiu contributed to the manuscript revision and final approval. All authors discussed the results and contributed to the final manuscript.
